# Literary Notes

**Published:** 1897-02

**Authors:** 


### Literary Notes.

The *Canadian Journal of Medicine and Surgery*, published at Toronto, made its appearance among our exchanges with the beginning of the new year. It is a very creditable first number and bespeaks the capability and enterprise of its editors. Its editorial staff consists of Drs. W. A. Young, J. J. Cassidy and E. Herbert Adams. It also announces a long list of department editors.

The *Medical Compend*, of Toledo, accentuated the close of its tenth year of publication by issuing, in December, 1896, an enlarged and illustrated number, containing portraits of its editorial staff and collaborators. It was an interesting edition, that bespoke the enterprise and prosperity of our excellent contemporary.

It is announced that the *Tri-State Medical Journal* and *The General Practitioner*, of St. Louis, will be consolidated under the name *Tri-State Medical Journal and General Practitioner*. Dr. James Mooers Ball, by purchase, has secured the control of both journals and will edit the magazine as consolidated.

The *American Medical Journalist* is the name of a new monthly that began its existence with the new year. It appears to be owned, edited and published by Charles Wood Fassett, Esq., the enterprising medical publisher, of St. Joseph, Mo., and the secretary of the American Medical Publishers' Association. The first number is an extremely handsome specimen of magazine making and it deserves to succeed, if conducted on the lines of this specimen. Its subscription price is $1.00 a year—no exchanges, no free list.

Late Personal.—Albany, Jan. 22.—The State Civil Service Commission announces the appointment of Dr. Percy Bryant, of Buffalo, as medical superintendent of the Manhattan State Hospital. Dr. Bryant is now first assistant physician at the Buffalo State Hospital.

